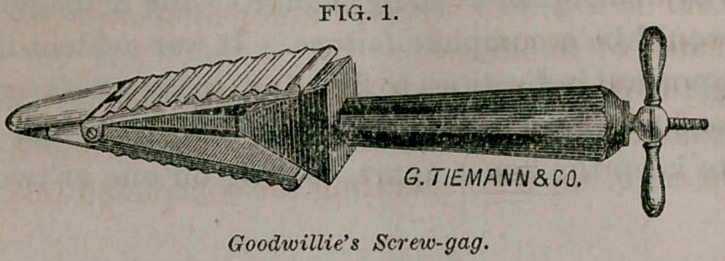# Immobility or Closure of the Jaw

**Published:** 1876-01

**Authors:** W. F. Westmoreland

**Affiliations:** Prof. of Surgery in the Atlanta Medical College


ATLANTA

MEDICAL AND SURGICAL JOURNAL.

VOL. XIII.] JANUARY—1876. [No. 10.

### Original Communications.

### IMMOBILITY OR CLOSURE OF THE JAW, WITH REPORT OF CASES.

BY W. F. WESTMORELAND, M.D., PROF. OF SURGERY IN THE ATLANTA MEDICAL COLLEGE.

*Read before the Atlanta Academy of Medicine.*

I propose in this paper to state briefly the more frequent causes producing closure or immobility of the inferior maxillary, and to illustrate each variety by the report of one or more cases, that have come under my observation.

I have been induced to select this subject for the essay required of me by the rules of the Academy, first, to demonstrate the possibility of a complete cure in a class of cases regarded by many as incurable, and for which Esmarck and others make a section of the jaw and an artificial joint, to relieve the sufferer of some of the troubles incident to the affection; and, secondly, for the reason that in some of our standard works on surgery this affection is entirely ignored, by others barely mentioned, and in no work does it receive that attention which its frequency, its distressing character when complete, and the difficulties attending its treatment, demand; and, thirdly, to present to the profession a screw-gag, which I had constructed some years ago, and which I feel fills the indication more perfectly than any instrument now in use.

The causes producing this trouble are various, and the treatment for its relief, as will be seen, will depend to a great extent upon the causes producing the accident. A frequent cause of immobility of the inferior maxillary is a chronic arthritis of the temporo-maxillary articulation. When the affection from this cause is well established the patient experiences as great difficulty in closing the jaw as in opening it.

Disease or injuries of the condyloid processes of the lower jaw, resulting in necrosis or caries, or what is more frequent, fracture of the condyles near the articulation, either with union in mal-position or excessive osseous deposits, often obstruct the movements of the bone, and if not properly attended to at the time, by making forcible movement, if necessary, will result in permanent closure of the inferior maxillary.

A spasmodic contraction of the muscles of mastication, the result of some irritation in the vicinity, as the cutting of a wisdom-tooth, sometimes produces closure of the jaw, as is strikingly illustrated in case No. 3.

One of the most frequent causes, however, in the cases that have come under my observation, and I believe it is the experience of others, is extensive sloughs, the result of profuse salivation. Fortunately this cause is not so frequent as a quarter of a century or more ago, when the administration of mercury to ptyalism was regarded, by many practitioners in the South and Southwest, as essential to the proper treatment of certain affections incident to their locality. While ptyalism is much the more frequent cause of sloughs in this locality, still we occasionally meet with such loss of tissue from other causes. But from whatever cause this loss of tissue, it is very frequently not confined to the soft parts covering the bones, with, perhaps, a loss of the alveola process by necrosis, but is often attended by extensive slough of the cheeks and lips, so that as the result we have the inferior and superior maxillary and the cheeks or lips or both firmly united by dense and unyielding nodular cicatricial tissue.

While there are other causes which may produce closure of the jaw, as tumors of the neck, tumors of either the superior or inferior maxillary, or in the vicinity of the temporo-maxillary articulation, etc., etc., still the varieties above mentioned embrace the cases that we are usually called upon to treat, and which are, to a greater or less extent, amenable to treatment. When the closure is complete, and the jaw, from whatever cause, is fixed, the subject is truly in a most pitiable condition. In such cases, if the front teeth are perfect, the sufferer has great difficulty in introducing proper nourishment, and is confined to such a diet as may be pressed through the irregularities of the teeth, or such as may be reduced to a fluid, or semi-fluid form—mastication being impossibe.

In the report of the following cases we have selected from our note-book such as may be regarded as the type of the particular form which they represent. We feel that in this way we can best illustrate the different forms or causes producing the trouble, the extent to which each is amenable to treatment, and the plan of treatment found most successful.

Of closure or immobility, the result of chronic arthritis of the temporo-maxillary, I have been consulted in five cases, three of which were rheumatic in character, and two traumatic, and directly traceable to an injury of the articulation.

In two of the cases, resulting from rheumatism, the jaw was fixed, but not completely closed. In the third there was complete closure. In all the cases rheumatic arthritis was general, all the articulation being more or less involved, and in many, complete immobility from fibrous and osseous deposits. No effort to break up adhesions in either case was made. Of the two traumatic, both submitted to treatment, with the happiest results. The following case illustrates this class of cases:

Miss W., fourteen years of age, consulted me three years ago for an affection of the jaw, which she said was the result of a blow (the kick of a horse if we recollect correctly) in the region of the temporo-maxillary articulation of the right side. Upon examination we found fibrous deposits, evidently the result of an inflammation of the articulation. The immobility was not complete. Motion to the extent of one-eighth of an inch was still preserved. After slightly separating the jaws, by means of a thin elevator, the screw-gag or speculum oris (Fig. 2) was insin-

FIG. 2.

*Westmoreland's Screw-gag.*

uated between the teeth, and, by a few turns of the screw, the adhesions were broken up, and the jaws separated to half their normal width, without the use of an anæsthetic, or excessive suffering to the patient. In three sittings, in as many days, I succeeded in opening the mouth to its normal width, and without inducing much inflammation of the articulation. Each time the mouth was opened she found it impossible to shut it by the action of the muscles, it being necessary to assist the muscles by pressing the jaws together. While she could not voluntarily close the jaws when extended to their full capacity, still she could, after the third sitting, open and close the mouth to the extent of half an inch. The instrument was introduced daily, and the jaws separated to their normal capacity; and in the interval she was instructed to use the jaws to the extent possible.

After remaining in the city for a few days, and learning to use the instrument, I permitted her to leave for her home in a distant part of the State, with the promise, however, that she would keep up the treatment, and return in a few weeks.

Two months elapsed before I saw her again, when I found her able to masticate her food to some extent, but she had gained little in the extent of the movements of the jaw. From the constant use of the instrument for a week or two after she left the city, the teeth had become necessarily sore to the extent of deterring her from its use, a week or more having elapsed since she had used it to the extent of opening the mouth to its normal capacity. To relieve this soreness, and to have the pressure equally distributed to all the teeth, I advised that she have her dentist construct a hard rubber plate, as suggested and used in Case No. 4, and continue the treatment as before suggested. Some months later I heard she was rapidly improving, and I suppose before this she is entirely relieved.

Where the obstruction to the movements of the inferior maxillary is the result of a lesion of the bone itself, it is, as above suggested, most frequently the condyloid processes that are involved. Sometimes, however, in lesions of the bodies of either the superior or inferior maxillary, as in some forms of fibro cystic tumors, there is almost complete immobility, as I have recently witnessed in two cases. In cases of necrosis of the condyles, whether circumscribed or involving the entire condyloid process, but little can be accomplished more than to keep up passive motion, however circumscribed, until the dead bone is eliminated, when every means possible should be used in extending the motion of the bone. Oftentimes it repays well the effort, as the results are of the most gratifying character. In other cases, however, but little can be accomplished, and the subject is doomed forever after to suffer the annoyance, or to submit, when practicable, to an operation for artificial joint.

But it is in injuries of the condyles, and particularly fractures of this portion of the bone, either with union in mal-position, or excessive osseous deposit in the vicinity of the articulation, that makes the class of cases that gives the surgeon most trouble. The following case is a striking illustration of the closure, or immobility, from this cause:

Miss C., when five years old, fell from the banisters to the floor below, a distance of twelve or fourteen feet, and fractured one condyle and dislocated the other. The dislocation was reduced, the fractured bone adjusted, the ordinary bandages for treating fractures of the inferior maxillary applied, and all went well for three or four weeks. When the bandage was removed, the fracture was found united, and the patient dismissed without a thought of anything unusual in her case. Ten days or two weeks later, her mother informed me that the movements did not return, as I had suggested; but if any change, the movements of the joint were more rigid for thc past few days than before. Upon examination, I found considerable rigidity; the jaw could not be opened by muscular action more than one-third its normal width, and to that extent, as the little patient contended, with more or less pain; so, there was an indisposition to use the jaw. Impressed with the idea that the union was not perfect, and that perhaps there was motion between the fragments, I made a careful examination and satisfied myself that the union was perfect. The provisional callus, at the point of fracture, which was easily detected when the bandage was removed, was still prominent. Feeling that the rigidity was dependent upon the provisional callus, in the vicinity of the articulation, and that this would be gradually absorbed and the movements of the bone restored, I quieted the fears of the mother by telling her that such would be the result in a few months. I also counselled her to encourage the child to use the jaw in every way possible, and to see that once in every twenty-four hours the jaw was opened to the extent possible by muscular contraction, and by means of a wedge or soft piece of pine, to slightly increase the movement by assisting the action of the muscles. Soon after this the family left the city, and I lost sight of the case. Three years later I was again consulted, and found the jaw in a very unsatisfactory condition. I learned that, instead of the expected improvement a few months after the injury, the movements became more restricted and more difficult, until at last the bone failed to respond to the action of the muscles and there was complete immobility. In this condition she was placed under the care of a surgeon in New York for a few months, who, with an instrument, opened the mouth, and in this way continued to treat her during her stay. In a few months after her return home, she was in the same condition as before the treatment was commenced in New York. I found that the osseous tumor, which was regarded as provisional callus, had greatly increased in size—perhaps three times the size it was when I saw her before. There was complete immobility. If the mouth was opened it would remain in the same position it was left, as the contraction of the muscles of mastication was not sufficient to close it. For several days in succession I opened and shut the mouth by means of the screw-gag with marked improvement in her condition. One of the great troubles in the treatment was to have the child submit to the frequent use of the instrument, and to use the jaw sufficiently often to keep up the improvement. I had constructed the hard rubber plates to save the teeth from the injurious effects of the instrument. I tried to impress the importance of perseverance in the treatment as the only hope of relief. If this plan was found ineffectual, or the child would no longer submit, I suggested Bean's apparatus for treating fractures of the inferior maxillar, so arranged that the jaw could be opened and shut by means of a screw. Whether my suggestions were adopted, or what has been the result, I am unable to say, as I have never seen the case since.

Partial immobility or rigidity of the jaw, from muscular contraction, is not very rare, but complete closure from tonic muscular contraction is not often met with. The following is the only case that has ever come under my observation.

Case III.—In 1860 I was called to see Mrs. D., twenty-five years old, who gave me the following history of her case: Ten weeks before my visit her trouble commenced with what she called a "jaw ache," which extended over the entire right side of the face and head, producing, as she expressed it, "a stiffness of the jaws." At the end of a week or ten days she was almost entirely relieved, a slight soreness only remaining, particularly just back of the last molar tooth of the right side. This continued, sometimes better for two or three days, and again for a few days worse, but upon the whole gradually growing worse. Four weeks after the commencement of her trouble, and six weeks before my visit, she rapidly grew worse, and in twenty-four hours the jaw was completely closed, so that the least movement was impossible. This condition, with more or less pain, had continued without interval up to my visit, or near six weeks. Various remedies had been administered, but nothing had succeeded in relieving the muscular contraction. For a month or more large doses of some preparation of opium had been daily administered to relieve her intense suffering. Upon examination I found the jaw slightly swollen in the region of the angle. The muscles of mastication were contracted to the extent that I had difficulty in introducing a knife blade between her teeth.

I decided at once that the whole trouble, reflex muscular contraction and all, was the result of either a wisdom-tooth passing through the soft parts, or a decayed tooth or fang. In either case the only hope of relief was the removal of the source of irritation. Chloroform, to complete anæsthesia, was administered, the jaw opened with a screw-gag, and a wisdom-tooth, right side, lower jaw, was discovered, with the gum covering it, greatly swollen and ulcerated. Free incissions were made, and after considerable difficulty the tooth was extracted.

There was but little or no disposition to a contraction of the muscles while the patient was under the influence of chloroform, but as soon as the effect passed off, the contraction returned, not, however, with the same force as before. Without further treatment, with the exception of an occasional anodyne, the patient steadily improved. At the end of a week she was able to partially open the mouth, and in two weeks had entirely recovered.

Closure or immobility of the jaw, the result of salivation, or gangrene and sloughs from other causes, I have met with more frequently than from all other causes combined. Many cases, after repeated operations, have been abandoned by surgeons as incurable; others have submitted to the operation suggested by Esmarck and Rizzoli, with the idea of partially remedying the defect by establishing an artificial joint.

Notwithstanding the unfavorable results of both the surgeons of Europe and America, yet I commence the treatment of this variety of closure with as much confidence as to the final favorable results as in any admitted curable affection requiring the same time and attention in its treatment. And yet I adopt the plan of treatment that many prominent surgeons, both in this country and Europe, contend is often worse than useless. In speaking of the treatment of this variety of closure of the jaw, Thomas Bryant, surgeon to Gay's Hospital, London, in his valuable work on the Practice of Surgery, says:

"When due to cicatrices and nodular plastic matter, little good has ever been derived from their division. Esmarck, of Kiel, in a paper 'On the Treatment of Closure of the Jaws from Cicatrices, 1860,' has, however, described an operation for the formation of a new joint in these cases, his operation consisting in a removal of a piece of the lower jaw. Mr. Henry and Mr. Heath, in this country, have both put into practice Esmarck's operation, and the success has been enough to indicate its great value. It must be mentioned that about the same year Rizzoli, of Boglogna, performed a somewhat similar operation to Esmarck's, dividing the jaw but not removing any portion.

" *Operation.*—This may readily be done by making an incision along the lower border of the jaw, in front of the masseter, and raising the integument, completing the operation by removing a wedge of bone, measuring about an inch above and half below, with a hand or chain saw. Where only one side of the jaw is affected, it is without doubt the best operation to be performed, the patients recovering their masticatory power in two or three weeks."

And this is all that he says in reference to the treatment of this class of cases, so that we can but infer that in every case he would be content to establish an artificial joint.

The following case is the type of this variety, and forcibly illustrates the cause of the many failures after a division of the cicatrical tissue uniting the bones, and suggests the plan which in my hands has ever been successful:

Master G., seven years old, from an adjoining county, was brought to my office by his father, who gave the following history: Three or four years before, during a severe illness, he was profusely ptyalised, from which he had a slough of the soft parts, covering the jaw bones of the right side; an extensive slough of the cheek, but not extending to the skin; a slight slough of the lip near the angle on right side. Two operations had been performed, each by dividing the cicatrix, and the jaw was opened half an inch or more, but in a short time returned to the position occupied before the operation. Upon examination, I found the jaw firmly closed by a cicatrix extending from the second incisor to the angle ot the jaw. The cheek and bones were apparently united by the same firm, dense, nodular tissue, so that there was not the least movement possible. The slough in the lip had filled by granulations, and was slightly attached by the same cicatrix. He was nourished by fluids and such solids as he could press between the irregularities of the teeth with his fingers. He was truly in a pitiable condition.

An operation was decided upon, and on the next day the patient was put partially under the influence of chloroform and the cicatrix uniting the bone was divided to the extent it could be reached. Assisted by a thin elevator and a spatula, after considerable difficulty the screw-gag was forced between the teeth, and the jaw opened to the extent of half an inch, thus putting the nodular tissue upon the stretch. The dissection was continued until the cheek was separated from the jaw and the jaws entirely separated. It became necessary during the operation to remove several pieces of this nodular tissue. The screw-gag was kept constantly between the teeth, and the screw turned from time to time that the tissues preventing the separation of the bones be put on the stretch and more readily incised. The jaws were separated to their normal width, as was demonstrated by comparing the extent of separation with that of a boy of his own age.

It was proposed to treat him now by keeping the mouth open fifteen hours in the twenty-four by blocks of soft pine and rubber. All did well for the first few days, or until the wound commenced granulating. In a few days later it was evident that the granulations were gaining on my blocks. The teeth became excessively sore from the constant pressure. From day to day it could be seen that the space between the jaws was lessening, as the teeth were so sensitive that they could not bear the pressure necessary to keep the jaw open. The soft parts covering the bones at the angle of the jaw were uniting, as well as the cheeks to the bones, so that it was evident, without some change in the management, my operation would be a complete failure. It was evident that there were two important indications to fill: first, to save the teeth, which had become loose and exceedingly sensitive from the pressure necessary to keep the bones apart, coming on one or two teeth at a time; and, secondly, to interpose something between the two granulating surfaces, which would make the union of the two surfaces impossible. Upon reflection, it occurred to me that both would be perfectly filled by the hard rubber plates, such as dentists use, minus the teeth—one for the upper and one for the lower jaw, accurately fitting the teeth, and thus distributing the pressure and extending back to the angle of the jaws, or to the depth of the incision. Thus we would have the raw surfaces of both jaws covered by a plate, making it impossible for the two granulating surfaces to unite. The slight flange covering the jaw could interpose between the cheek and jaw and thus prevent their union. I determined to test it. The patient was allowed to rest for forty-eight hours, until the extreme sensitiveness of the teeth subsided. Having everything in readiness, chloroform to partial anæsthesia was administered, and the jaws separated and all adhesions that had taken place severed with a bistoury. As soon as the hemorrhage ceased, Dr. Charles D'Alvigny, whom I had engaged to make the plates, took a cast of both the superior and inferior maxillary. The plates were made of hard rubber, and fitted accurately. The mouth was kept open, when desired, with a block of soft rubber, introduced between the plates. I had no further trouble with the teeth, or with the case. The plates were removed once or twice every twenty-four hours, for the first week or two, and cleansed and put back. In a short time my little patient learned to remove and readjust the plates himself. I furnished him with a little block, which, when introduced in a certain position, separated the jaws to their normal capacity. This was introduced, for a short time, two or three times every day. Ten years have now elapsed since the operation, and no one, after a careful examination, would suspect from appearances that he had ever had closure of the jaw. The movements of the inferior maxillary are as free as any one of his age, and, with the exception of the cicatrix from the slough of the lip, there is not the least deformity. Since treating this case, I have treated three others, and with like success.

One word in regard to a new screw-gag or speculum oris, and I am done. All the screw-gags that I have ever seen, until I had one constructed, (Fig. 2), opened obliquely. Thus Dr.

FIG. 1.

*Goodwillie's Screw-gag.*

Goodwillie's instrument (Fig. 1) opens by a screw in one end, and in opening the mouth, if it requires great force, will displace the teeth inwards. Some of them open with a screw, but the blades open like a pair of scissors—obliquely outwards—and will invariably displace the teeth outwards. Before having constructed my instrument (Fig. 2) I frequently displaced the teeth, breaking off some and forcing out others. In my instrument, by turning the screw, the blades separate parallel with each other, so that we always have the pressure in the direction of the axis of the tooth.

## Figures and Tables

**Fig. 2. f1:**
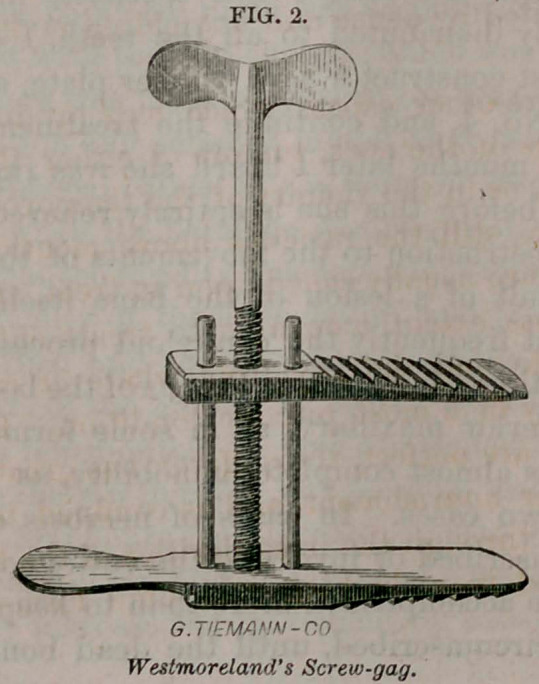


**Fig. 1. f2:**